# Supplementary material for: Genome-Wide Identification of Arachis hypogaea LEC1s, FUS3s, and WRIs and Co-Overexpression of AhLEC1b, AhFUS3b, AhWRI1a and AhWRI1d Increased Oil Content in Arabidopsis Seeds
Source: Plants (Basel). 2025 Sep 19;14(18):2910. doi: 10.3390/plants14182910 (PMC12473564; doi:10.3390/plants14182910)
Supplement: Supplementary file 1 [file plants-14-02910-s001.zip › Supplementary Figures and legends.docx]

**
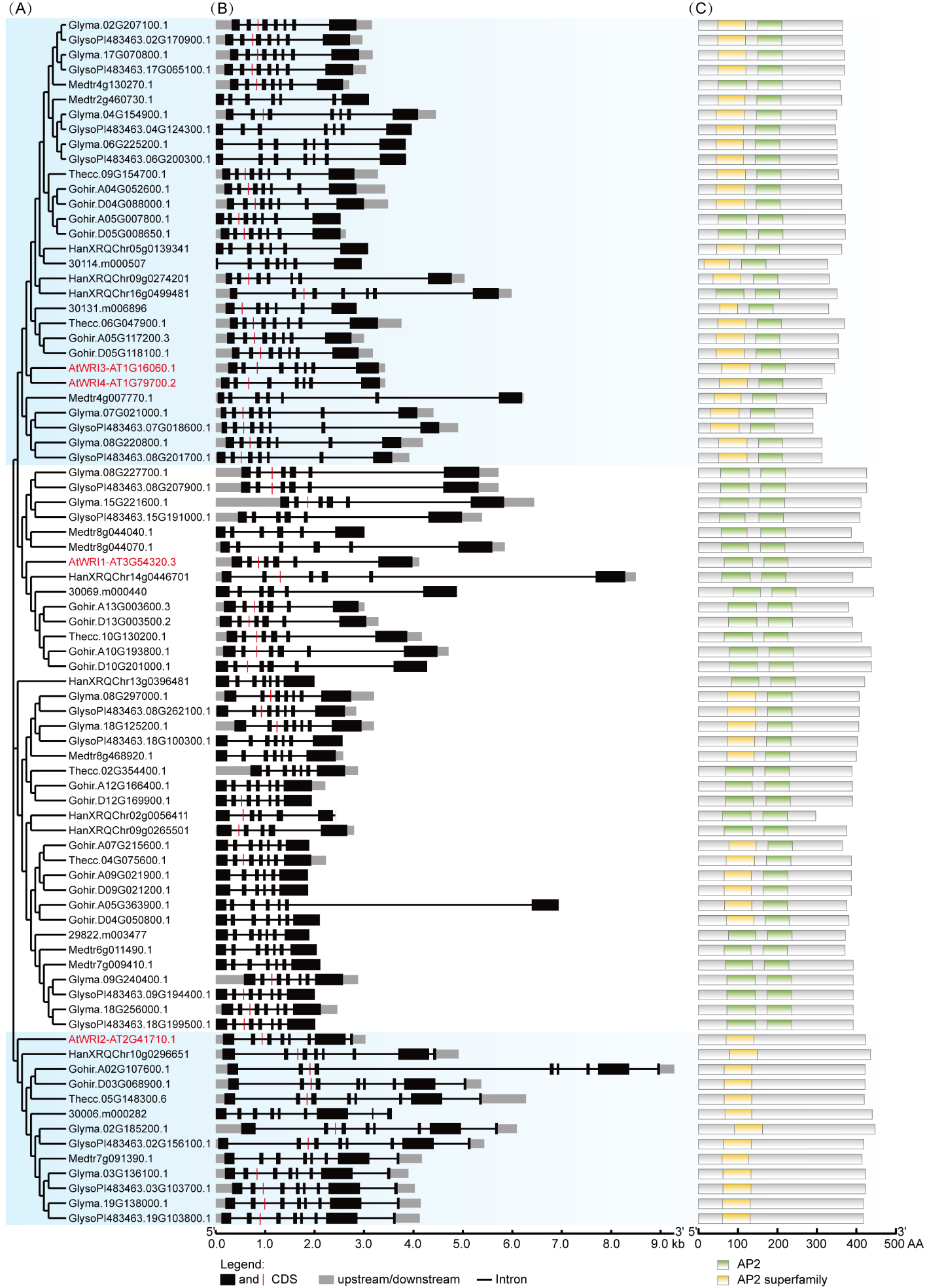
**

**Figure S1.** Phylogenetic relationships, gene structures, and conserved domains of the WRI family members in other plant species.

(**A**) Phylogenetic tree. Protein sequences were used to construct the phylogenetic tree using the Maximum Likelihood method with Jones-Taylor-Thornton (JTT) model of MEGA11 software [53]. Proteins from *Arabidopsis* are highlighted in red.

(**B**) Gene structures. Genomic DNA and coding sequences were used to construct gene structures via the Gene Structure Display Serve (GSDS2.0; <https://gsds.gao-lab.org/>, accessed on 20 October 2024) [54]. Black boxes denote exons within coding regions, lines represent introns, and thin red boxes mark the conserved 9-bp micro-exons (encoding VYL; Figure 2C-D and Figures S4-6). Gray boxes indicate untranslated upstream or downstream regions. The lengths of boxes and lines represent correspond to the size of exons and introns, respectively.

(**C**) Conserved domains. A functional search of conserved domains was performed using full-length protein sequences via Batch CD-Search with default parameters in the Conserved Domain Database (CDD) in NCBI (<https://www.ncbi.nlm.nih.gov/Structure/bwrpsb/bwrpsb.cgi>, accessed on 22 October 2024) [55]. Corresponding sequences in plant species were obtained from their respective genome databases (Table S1). All WRI1s, WRI3s, and WRI4s contain two AP2 domains (smart00380 and cl00033 for AP2 and AP2 superfamily, respectively), while all WRI2 homologues have only one AP2 domain (smart00380).

**
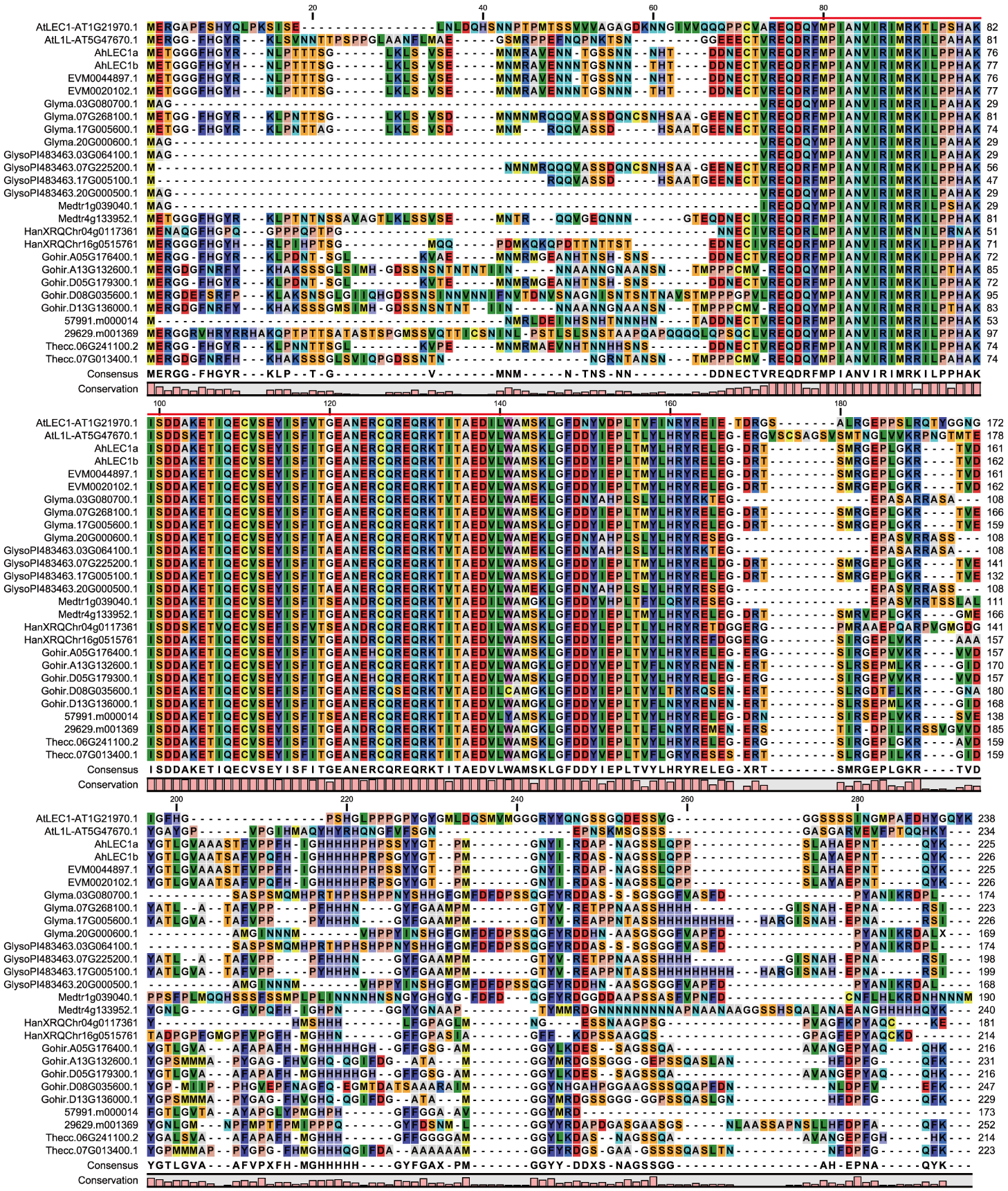
**

**Figure S2.** Protein alignment of AhLEC1s and their homologs.

The central B domains of AhLEC1a (amino acids 52–141) and AhLEC1b (amino acids 53–142) exhibited strong similarity to those of other plant LEC1s. In contrast, low sequence similarity was observed in the amino- and carboxyl-terminal regions among AhLEC1s and other LEC1 homologs. The conserved B domain is indicated by a red line. Numbers on the right indicate the end positions of amino acid residues within each protein; numbers above the alignment serve as reference points. In the consensus line, uppercase letters and “X” indicate residues conserved in more and less than 50% identity in the sequences, respectively.

**
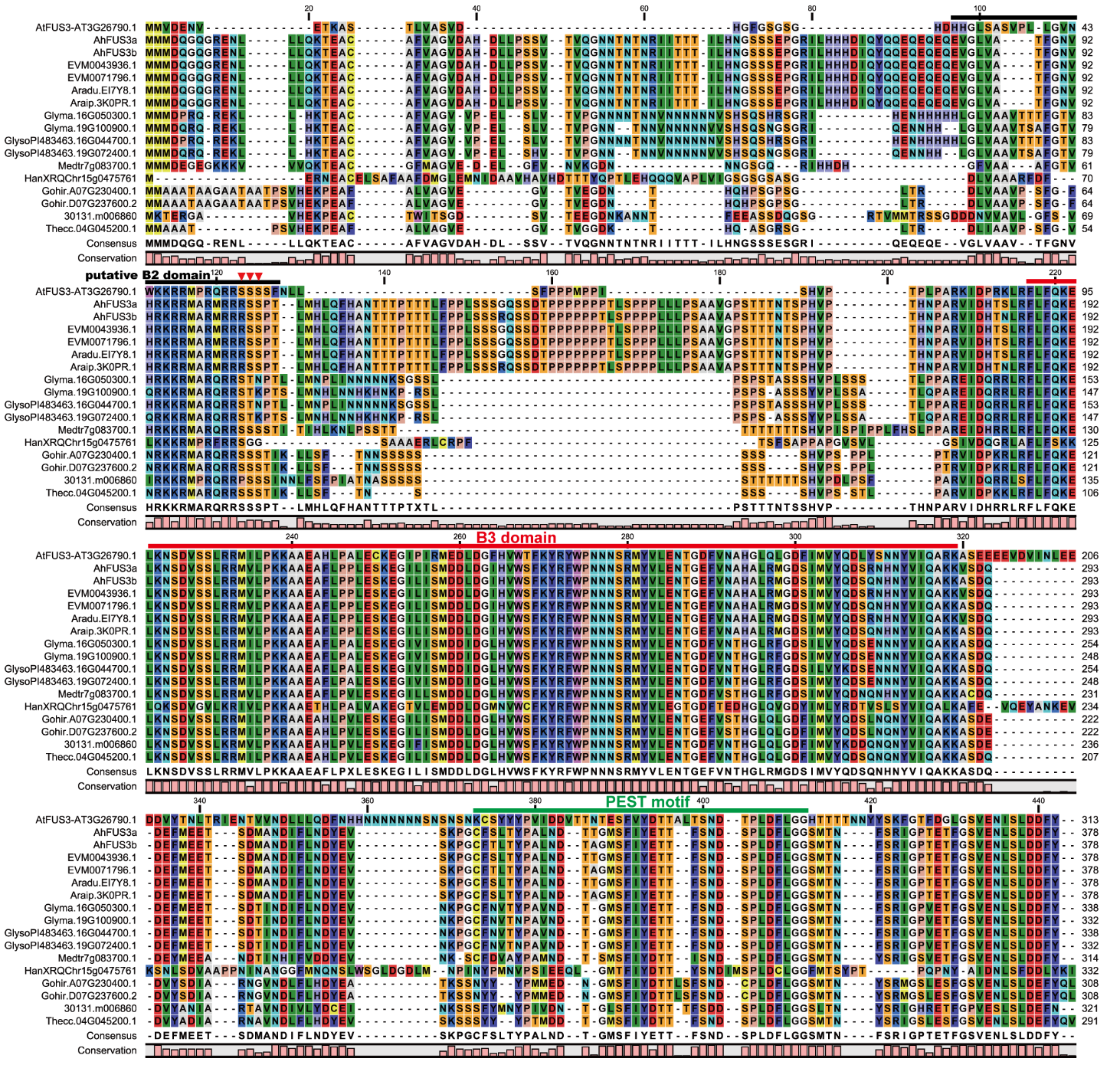
**

**Figure S3.** Protein alignment of AhFUS3s and their orthologs.

Putative B2 domains and B3 domains are indicated by black and red lines, respectively. Putative NLSs (Table S8) are located within the putative B2 domains, although deletion of the B2 domain in AtFUS3 does not affect nuclear localization [62]. Three red triangles denote the serine residues (55-SSS-57) in the N-terminal domain of AtFUS3, which are phosphorylated by AKIN10 to promote protein instability [59]; these sites are not fully conserved among plant FUS3s. A green line marks the PEST motif in the C-terminal domain, which enhances AtFUS3 degradation via the 26S proteasome pathway [62]. One or two putative PEST sequences were identified in *A. hypogaea* and other plant FUS3s (Table S8). Numbers on the right indicate the end positions of amino acid residues within each protein; numbers above the alignment serve as reference points. In the consensus line, uppercase letters and “X” indicate residues conserved in more and less than 50% identity in the sequences, respectively.

**
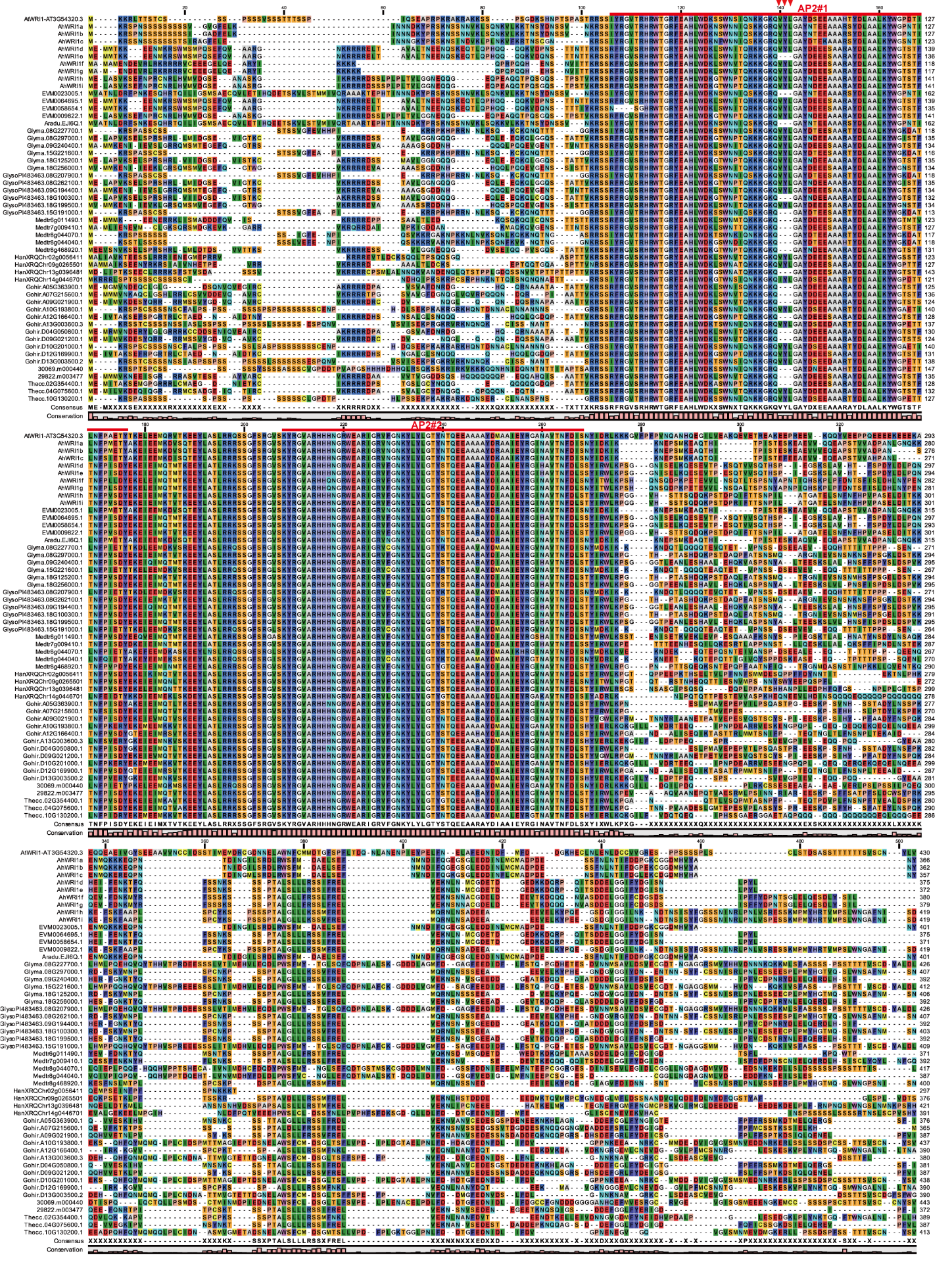
**

**Figure S4.** Protein sequence alignment of AhWRI1s and their homologs.

The two conserved AP2 domains, designated AP2#1 and AP2#2, are highlighted with red lines. The VYL motifs located within AP2#1 are marked with red triangles. Numbers on the right indicate the end positions of amino acid residues within each protein; numbers above the alignment serve as reference points. In the consensus line, uppercase letters and “X” indicate residues conserved in more and less than 50% identity in the sequences, respectively.

**
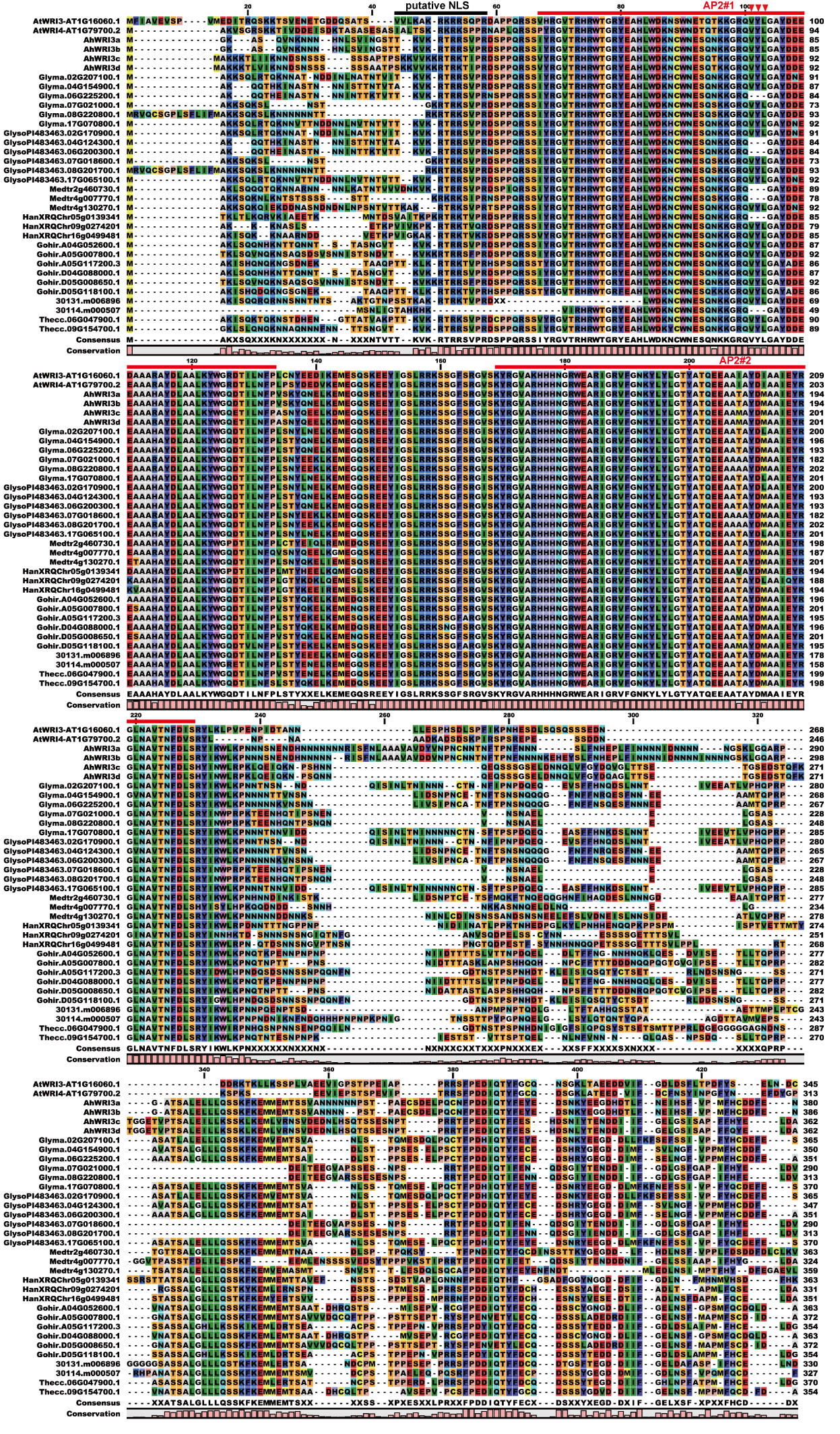
**

**Figure S5.** Protein sequence alignment of AhWRI3s and their homologs.

The two conserved AP2 domains, designated AP2#1 and AP2#2, are highlighted with red lines. The VYL motifs located within AP2#1 are marked with red triangles. Black lines denote the putative NLSs (Table S8). Numbers on the right indicate the actual positions of the final amino acid in each sequence; numbers above the alignment serve as reference points in the text. In the consensus line, uppercase letters represent residues identical in more than 50% of the sequences, and “X” indicates positions with less than 50% identity.

**
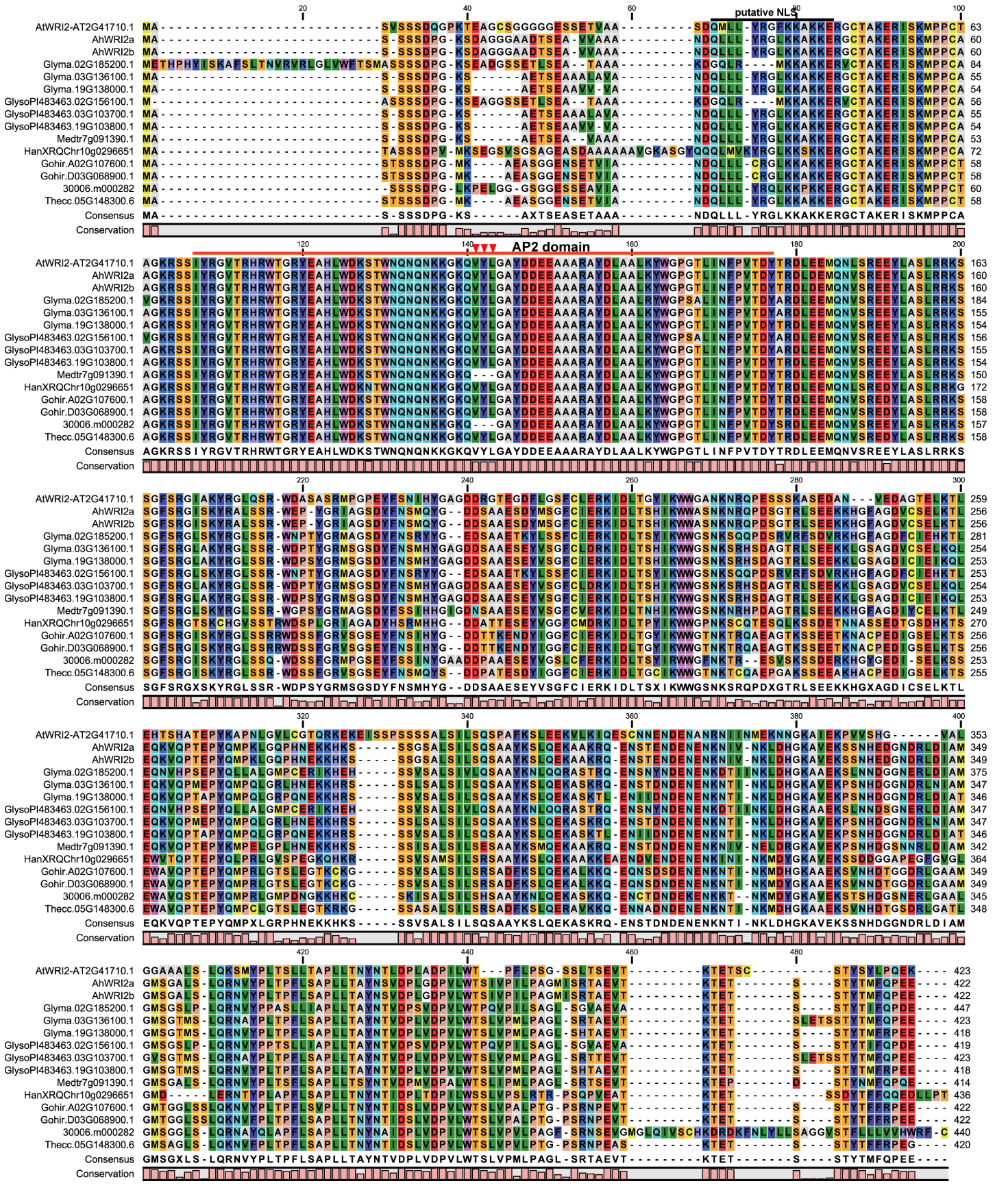
**

**Figure S6.** Protein sequence alignment of AhWRI2s and their homologs.

Red triangles indicate the “VYL” motif. Red and black lines mark the AP2 domain and the putative NLSs (Table S8), respectively. Numbers on the right indicate the actual positions of the final amino acid in each sequence; numbers above the alignment serve as reference points in the text. In the consensus line, uppercase letters represent residues identical in more than 50% of the sequences, and “X” indicates positions with less than 50% identity.

**
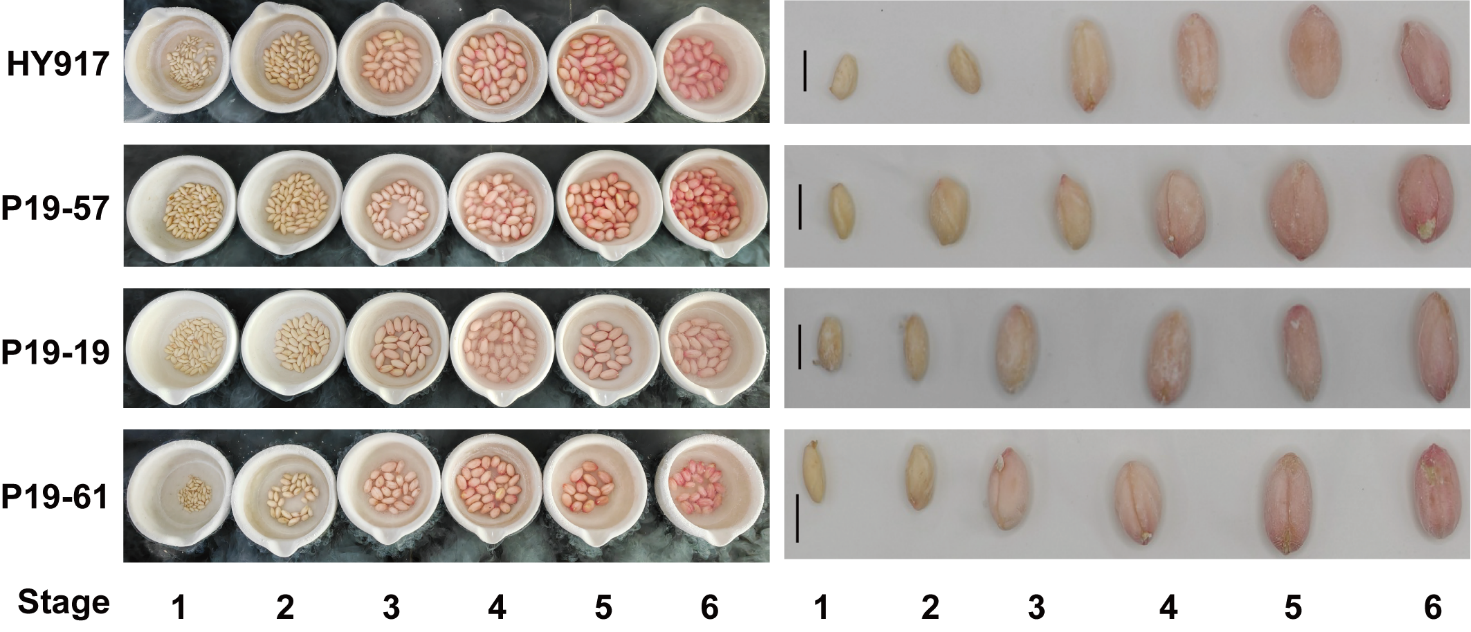
**

**Figure S7.** Seed developmental stages of four peanut sister lines (HY917, P19-19, P19-61, and P19-57).

Seeds were categorized into six developmental stages based on kernel morphology according to Pattee et al. (75):

**Stage 1 (Pattee 4):** very small, flat, entirely white.

**Stage 2 (Pattee 5):** small and flat; white or just turning pink at one end.

**Stage 3 (Pattee 6/7):** torpedo shaped; pink at the embryonic axis end, white to light pink at the other.

**Stage 4 (Pattee 8):** entirely light pink.

**Stage 5 (Pattee 9):** dark pink at the embryonic axis end, light to dark pink elsewhere.

**Stage 6 (Pattee 10):** large, generally dark pink throughout; seed coat beginning to desiccate.

Three biological replicates were analyzed per stage, each consisting of at least 10 seeds. Scale bar =1 cm.

**
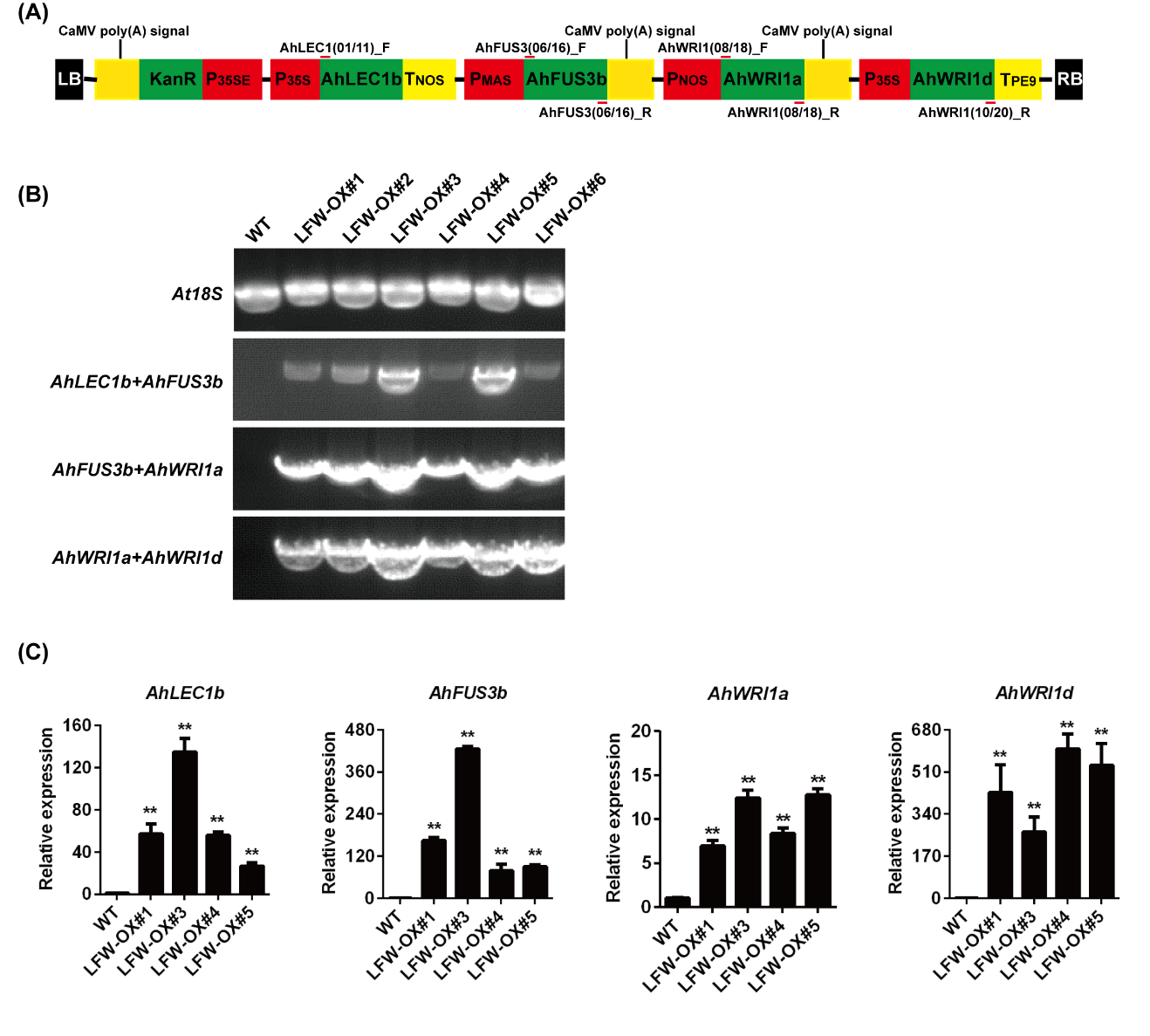
**

**Figure S8.** PCR detection of transgenic lines.

(**A**) Schematic diagram illustrating the primer pairs (Table S2) used for PCR detection. The primer pair AhLEC1(01/11)_F and AhFUS3(06/16)_R amplifies *AhLEC1b* and *AhFUS3b*; AhFUS3(06/16)_F and AhWRI1(08/18)_R amplifies *AhFUS3b* and *AhWRI1a*; AhWRI1(08/18)_F and AhWRI1(10/20)_R amplifies *AhWRI1a* and *AhWRI1d*. Short red lines indicate primer positions.

(**B**) PCR confirmation of six independent kanamycin-resistant transgenic plants using the three primer pairs and genomic DNA extracted from leaves.

(**C**) qRT-PCR analysis of *AhLEC1b*, *AhFUS3b*, *AhWRI1a*, and *AhWRI1d* expression in transgenic *Arabidopsis* lines LFW-OX#1, #3, #4, and #5. RNA was isolated from 15-DAF siliques. For samples with undetectable expression (Ct undetermined), the maximum cycle number (40) was used to estimate the upper limit of relative expression. *AtActin* was used as the internal control, and relative expression levels were calculated using the 2^-ΔΔCt^ method. Data are presented as mean ± SD (*n*=3 biological replicates). **p* < 0.05, ***p* < 0.01 versus WT (one-way ANOVA followed by Dunnett's test).

**Figure S9.** Composition analysis of unsaturated fatty acids, saturated fatty acids, C18 and C20, and ratios of unsaturated/saturated and C18/C20.

Comparison of the contents of unsaturated fatty acids, saturated fatty acids, C18 and C20 (**A**), and the ratios of unsaturated to saturated fatty acids and C18 to C20 (**B**) in dry mature seeds of wild-type and transgenic plants. Asterisks indicate significant differences between wild-type and transgenic plants (one-way ANOVA followed by Dunnett's test: **p* ≤ 0.05; ***P* ≤ 0.01). Values represent means ± SD (*n* = 3).

**

**

**Figure S10.** *In silico* expression analysis of *AtLEC1*, *AtL1L*, *AtFUS3*, *AtWRI1*, *AtWRI2*, *AtWRI3*, and *AtWRI4*.

Data are obtained from the Arabidopsis eFP browser (<https://bar.utoronto.ca/efp/cgi-bin/efpWeb.cgi>, accessed on 12 June 2024) [143].

**
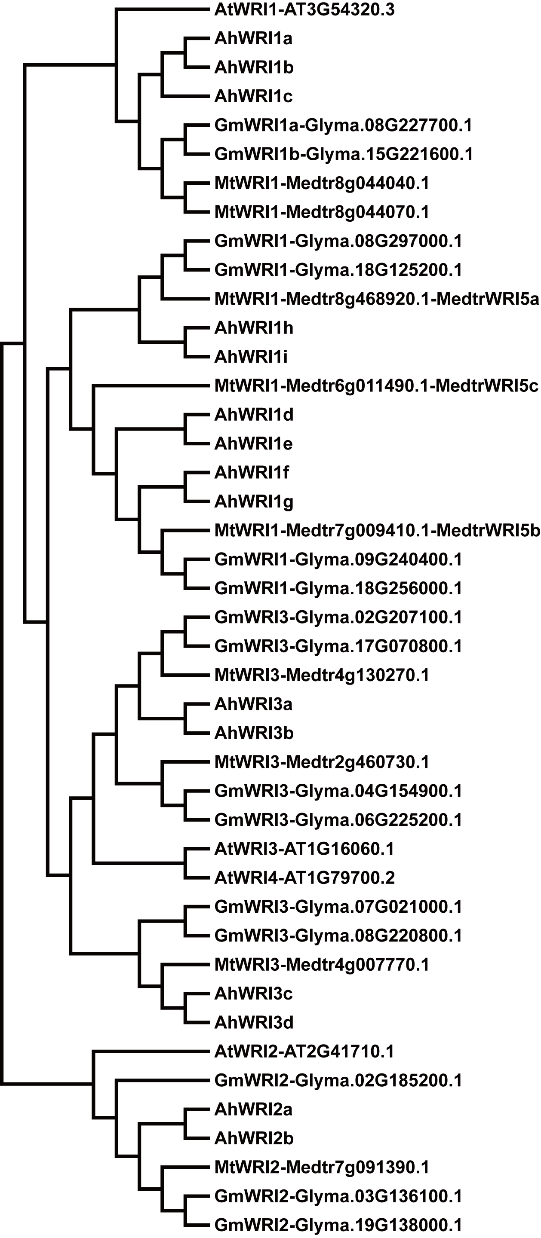
**

**Figure S11.** Phylogenetic analysis of WRI proteins from *A.* *hypogaea*, *A. thaliana*, *G. max*, and *M. truncatula* WRIs.

The Maximum Likelihood tree was constructed in MEGA11 using the JTT model [53]. Protein sequences of *A. hypogaea* were identified from HY917 (Table S2), while those of *A. thaliana*, *G. max*, and *M. truncatula* sequences were retrieved from their respective genome databases (Table S1).
